# Lysophosphatidic Acid Receptor 3 Suppress Neutrophil Extracellular Traps Production and Thrombosis During Sepsis

**DOI:** 10.3389/fimmu.2022.844781

**Published:** 2022-04-07

**Authors:** Shengqiang Pei, Chuansheng Xu, Jianqiu Pei, Ruifeng Bai, Rui Peng, Tiewei Li, Junjie Zhang, Xiangfeng Cong, Jerold Chun, Fang Wang, Xi Chen

**Affiliations:** ^1^ State Key Laboratory of Cardiovascular Disease, Fuwai Hospital, National Center for Cardiovascular Diseases, Chinese Academy of Medical Sciences and Peking Union Medical College, Beijing, China; ^2^ The Key Laboratory for Cell Proliferation and Regulation Biology of Ministry of Education, College of Life Sciences, Beijing Normal University, Beijing, China; ^3^ Neuroscience Drug Discovery, Sanford Burnham Prebys Medical Discovery Institute, La Jolla, CA, United States; ^4^ Diagnostic Laboratory Service, Fuwai Hospital, Chinese Academy of Medical Sciences and Peking Union Medical College, Beijing, China; ^5^ Department of Clinical Laboratory, Fuwai Yunnan Cardiovascular Hospital, Kunming, China

**Keywords:** thrombosis, sepsis, monocyte, lysophosphatidic acid receptor 3, neutrophil extracellular traps

## Abstract

Sepsis consists of life-threatening organ dysfunction resulting from a dysregulated response to infection. Recent studies have found that excessive neutrophil extracellular traps (NETs) contribute to the pathogenesis of sepsis, thereby increasing morbidity and mortality. Lysophosphatidic acid (LPA) is a small glycerophospholipid molecule that exerts multiple functions by binding to its receptors. Although LPA has been functionally identified to induce NETs, whether and how LPA receptors, especially lysophosphatidic acid receptor 3 (LPA_3_), play a role in the development of sepsis has never been explored. A comprehensive understanding of the impact of LPA_3_ on sepsis is essential for the development of medical therapy. After intraperitoneal injection of lipopolysaccharide (LPS), *Lpar3* ^-/-^mice showed a substantially higher mortality, more severe injury, and more fibrinogen content in the lungs than wild-type (WT) mice. The values of blood coagulation markers, plasma prothrombin time (PT) and fibrinogen (FIB), indicated that the *Lpar3* ^-/-^ mice underwent a severe coagulation process, which resulted in increased thrombosis. The levels of NETs in *Lpar3* ^-/-^ mice were higher than those in WT mice after LPS injection. The mortality rate and degree of lung damage in *Lpar3* ^-/-^ mice with sepsis were significantly reduced after the destruction of NETs by DNaseI treatment. Furthermore, *in vitro* experiments with co-cultured monocytes and neutrophils demonstrated that monocytes from *Lpar3* ^-/-^ mice promoted the formation of NETs, suggesting that LPA_3_ acting on monocytes inhibits the formation of NETs and plays a protective role in sepsis. Mechanistically, we found that the amount of CD14, an LPS co-receptor, expressed by monocytes in *Lpar3* ^-/-^mice was significantly elevated after LPS administration, and the MyD88-p65-NFκB signaling axis, downstream of toll-like receptor 4 signaling, in monocytes was overactivated. Finally, after an injection of the LPA_3_ agonist (2S)-1-oleoyl-2-methylglycero-3-phosphothionate (OMPT), the survival rate of mice with sepsis was improved, organ damage was reduced, and the production of NETs was decreased. This suggested the possible translational value and application prospects of (2S)-OMPT in the treatment of sepsis. Our study confirms an important protective role of LPA_3_ in curbing the development of sepsis by suppressing NETs production and thrombosis and provides new ideas for sepsis treatment strategies.

## Introduction

Sepsis is a life-threatening condition caused by a dysregulated host response to microbial infection, resulting in vascular dysfunction, shock, and organ failure. Sepsis remains one of the leading causes of death in humans and animals (1–3). Although inflammation is a central event in sepsis-induced death, increasing experimental and clinical evidence has indicated that pathological coagulopathy in sepsis leads to multiple organ dysfunction and is associated with increased mortality and morbidity (3–5).

During sepsis, excessive cytokine releases due to systemic inflammation results in the activation of the coagulation system. Ongoing activation of coagulation pathways during sepsis can overwhelm the anticoagulant system, leading to excessive intravascular thrombosis. Although many clinical trials have explored the use of drugs designed to attenuate inflammatory and/or coagulation pathways, most of them are not effective (6, 7). Thus, it is imperative to investigate the development of sepsis and to find more effective treatment strategies and therapeutic agents.

Neutrophils, the first line of defense in innate immunity during sepsis (8), protect the body from infection, and promote tissue repair through their phagocytic and antimicrobial activities (9). Neutrophil extracellular traps (NETs) are extracellular DNA bers comprising histones and neutrophil antimicrobial proteins (10). Different stimuli activate neutrophils to expel nuclear chromatin to form NETs, including pathogens and bacterial toxins (11–15), other cytokines (16–19), complement proteins (20, 21), and activated platelets (16, 22–24). Although NETs play a benecial role in defense against pathogenic infections by capturing and killing bacteria (10, 25), excessive NETs formation during sepsis has been documented to influence the dynamics of thrombus formation in several ways (26–31). Increased NETs lead to increased intravascular thrombosis and disseminated intravascular coagulation (DIC), contributing to organ damage, and increasing morbidity and mortality (26, 29, 32–35). Therefore, the inhibition of thrombosis by modulating the production of NETs could be a possible treatment of sepsis.

Lysophosphatidic acid (LPA) is a bioactive lipid mediator of inflammation *via* lysophosphatidic acid receptors 1–6 (LPA_1_–LPA_6_), contributing to the pathogenesis of many diseases including asthma, acute lung injury, fibrosis, and postnatal heart regeneration (36–38). A study reported LPA_1_ knockout reduces LPS-induced inflammatory cytokine signaling in the lungs (39), and inflammatory cytokines are effective inducers of NETs. Recently, we found that LPA regulates thrombotic stability by inducing NETs release from neutrophils *in vitro* (40).Thus, LPA receptors might be involved in influencing NETs release and coagulation processes during sepsis. However, some of the LPA receptors, such as LPA_3_, have not been associated with infection-induced immunity. In this regard, we investigated whether LPA_3_ connected NETs release was associated with thrombosis during sepsis and whether treatment with LPA_3_ agonist, such as (2S)-1-oleoyl-2-methylglycero-3-phosphothionate (OMPT) could improve sepsis outcomes. The roles and mechanisms of LPA_3_ in the complex pathophysiological processes of sepsis were explored in the present study with the hopes of identifying new mechanisms for the development of sepsis, and to provide new therapeutic strategies.

## Materials and Methods

### Animals

*Lpar3* ^-/-^ mice were gifts from Professor Jerold Chun (41), Wide-type (WT) littermates were served as controls. All the breeding colony was bred and housed in a specific pathogen-free barrier facility maintained by the State Key Laboratory of Cardiovascular Disease. The genotypes were verified by PCR using primer sets in **Supplementary Table 1**. All animal experiments were approved by the Laboratory Animal Management and Use Committee of Fuwai Hospital, Chinese Academy of Medical Sciences (animal application approval number FW-2019-0009). The investigation conforms to the Guide for the Care and Use of Laboratory Animals published by the US National Institutes of Health (NIH Publication No. 85-23, revised 1996) and the ‘Regulation to the Care and Use of Experimental Animals’ of the Beijing Council on Animal Care (1996).

### Mouse Model

For LPS-induced sepsis model, 8-week-old male *Lpar3* ^-/-^ and WT littermate mice with an average weight of 20–25 g was injected intraperitoneally with LPS (7 mg/kg, Escherichia coli 055: B5, Sigma-Aldrich, L2880) or an equal volume of phosphate-buffered saline (PBS) as a control. Survival rate was recorded every 4 hours for a 64 hours period after LPS injection.

For Cecal ligation puncture (CLP) sepsis model, 8-week-old male *Lpar3* ^-/-^ and WT littermate with an average body weight of 20-25 g were prepared and removed hair by using hair removal cream one day before the experiment. The mice were anesthetized with isoflurane and operated under isoflurane maintenance. By avoiding blood vessels, the abdominal skin and muscles of the mice were carefully incised (approximately 1 cm), the mouse cecum was slowly clamped out by reaching into the abdominal cavity with sterilized forceps, 50% of the cecum was ligated with 6-0 silk, and a 21G needle was used to puncture near the ligature (two holes). The ligated appendix was carefully returned to the abdominal cavity and the abdominal muscles and skin were sutured separately. After awakening, the mice were placed back in their cages and kept in a 12-hour fast. Sham-operated mice underwent the same procedure without ligation and puncture of the exposed cecum. Survival rate was recorded every 6 hours for a 96 hours period after CLP.

For DNase I-treated mouse model, DNaseI (Roche, 11284932001) was dissolved into a 20 U/g solution with PBS, and injected through the tail vein at 10 μL/g at 1-hour post-LPS-injection.

For (2S)-OMPT treated mouse model, 8-week-old male WT mice were injected intraperitoneally with LPS (15 mg/kg, Escherichia coli 055: B5, Sigma-Aldrich L2880), At 1-hour after LPS injection or CLP, (2S)-OMPT (10 mg/kg, Sigma-Aldrich, 857235P.) was dissolved in 3% (w/v) BSA/PBS, and injected through the tail vein. Survival rate was recorded every 4 hours for a 64 hours period after LPS injection or CLP.

### Lung Injury Measurement

Murray score was used to evaluate lung injury (42). The scale includes 4 items, each item scores 4 points, a score of 0 signified no injury, a score of 1 signified injury in 25% of the lung, a score of 2 signified injury in 50% of the lung, a score of 3 signified injury in 75% of the lung, and a score of 4 signified injury throughout the lung. The higher the score, the more serious the lung injury. The individuals who graded the severity of lung injury were blinded to the genotype.

### Myeloperoxidase (MPO)-DNA Complex Measurement

MPO-DNA complexes were identified using a capture ELISA. As the capturing antibody, 5 μg/mL anti-MPO polyclonal antibody (Merck Millipore; 07-496-I) was coated to 96-well microtiter plates overnight at 4°C. After blocking in incubation buffer, 40 μL of mouse plasma or culture supernatants was added per well in combination with the peroxidase-labelled anti-DNA monoclonal antibody (component NO.2 of cell death detection ELISA kit; Roche, CatNo:11774425001) following the manufacturer’s instructions. After 2 hours of incubation at room temperature (RT) on a shaking device, the samples were washed. 100 μL ATBS (one piece of component NO.7 added to 5 ml component NO.6) was added per well and incubation at 37°C in the dark for 25 minutes, 100 μL ATBS stop buffer (component NO.8) was added per well to terminate the reaction. The absorbance at 405 nm wavelength was measured by the automatic enzyme mark analyzer (Infinite_M2000, TECAN, Switzerland).

### Ds-DNA Measurement

Concentration of ds-DNA was measured using the *Quant*-*iT™ PicoGreen^®^* dsDNA Reagent and Kits (Invitrogen, P7589) according to the manufacturer’s instructions.

### LPA Measurement

LPA levels in mouse plasma were quantified by an enzyme-linked immunosorbent assay (ELISA) kit (USCN Life Science, CEK623Ge), according to the manufacturer’s instructions.

### Cytokine Measurement

IL-1β, IL-6, IL-8 detection kits customized from Bio-Rad (Bio-Rad, 17001201) were used to determine the cytokine levels in mouse plasma by Luminex technology according to the manufacturer’s instructions.

### Immunohistochemistry Staining

Lungs from control and LPS-challenged mice were embedded in paraffin, sectioned (5 μm), and mounted on glass slides. After dewaxing, antigen retrieval was performed using citrate buffer and then permeabilized with 0.1% Triton X-100 for 10 minutes. After being blocked by hydrogen peroxide, the slides were immunostained with Anti-Fibrinogen antibody (1:100, Abcam, ab34269), overnight at 4°C and rewarmed at 37°C for 30 minutes. Subsequently, sections were incubated with ready-to-use undiluted secondary antibodies conjugated with HRP for 30 minutes at 37°C. Subsequently, Diaminobenzidine (DAB) staining was used for 5 minutes at room temperature and the nuclei were stained with hematoxylin for 2.5 minutes at room temperature. The stained sections were observed under a light microscope. Images were analyzed using the Image-J software.

### Immunofluorescence Staining

The slicing preparation process is described above. After dewaxing, antigen retrieval was performed using citrate buffer and permeabilized with 0.1% Triton X-100 for 10 min, then specimens were blocked with goat serum. The sections were incubated with primary antibodies, specifically anti-citrullinated-histone H3 (1:100; Abcam; ab5103) and Anti-Myeloperoxidase antibody (1:200; Abcam; ab208670), followed by detection with the samples were incubated with Alexa Fluor-594-coupled (1:500, Invitrogen; A-11012) and/or Alexa Fluor-488-coupled (1:500, Invitrogen; A-11034) secondary antibodies overnight at 4°C. Coverslips were mounted with a VectaShield medium containing DAPI to detect DNA. The sections were imaged using a Zeiss inverted fluorescence microscope (AXI0; Zeiss) that was equipped with a Zensoftware or using a laser-scanning confocal microscope (SP8; Leica) with a 20×water immersion objective. Images were analyzed using the Image-Pro Plus 6.0 software (Media Cybernetics, Inc. Rockville, MD, USA).

### Microcirculatory Perfusion Detection

To determine microcirculatory perfusion during abdomen, mice were removed hair by using hair removal cream one day before the experiment. Microcirculatory perfusion was monitored when the mice were anesthetized and in a state of uniform respiration, abdomen was scanned using laser Doppler flowmetry (Perimed, Sweden) for 5 min.

### Mouse Blood Coagulation Assay

The prothrombin time (PT) and fibrinogen (FIB) was measured by using SF-400 semi-automatic coagulometer (Beijing Success Technology Inc) according to the manufacturer’s instructions.

### Mouse Neutrophils and Monocytes Isolation

Neutrophils were isolated using Neutrophil isolation kit (Solarbio, P9201) according to the manufacturer’s instructions. The purity of the neutrophil preparations (consistently > 95%) was routinely verified with Giemsa staining, and cell viability (>97%) was verified by trypan blue exclusion assay. Monocytes were isolated using a mouse monocyte isolation kit (STEMCELL Technologies, 19861) according to the manufacturer’s instructions.

### Detection of NETs Released by Plasma-Induced Neutrophils

Isolated neutrophils were determined and counted by hemocytometer and diluted to 10^5/L, SYTOX Green Nucleic Acid Stain (Thermo Fisher Scientific, S7020) was added to isolated neutrophils with final concentration of 5 μM. Take a 96-well plate (black walls and white bottoms) and spread into premixed neutrophils. added 50 μL plasma to the corresponding wells and incubated at 37°C for 4 hours. Measured the fluorescence of each well at 520 nm by a microplate reader and imaged using a Zeiss inverted fluorescence microscope (AXI0; Zeiss) that was equipped with a Zensoftware.

### Detection of NETs Released by Monocyte-Induced Neutrophils

Neutrophil pretreatment procedure is described above. Take a 96-well plate (black walls and white bottoms) and spread into premixed neutrophils. Added 50 μL isolated monocytes premixed with LPS (final concentration 0.5 mg/mL) to the corresponding wells and incubated at 37°C for 8 hours. Measured the fluorescence of each well at 520nm by a microplate reader and imaged using a Zeiss inverted fluorescence microscope (AXI0; Zeiss) that was equipped with a Zensoftware.

### Quantitative RT-PCR (qRT-PCR)

The total RNA was extracted from monocytes isolated from septic model by using TRIzol^®^ RNA isolation kit (Invitrogen; 15596026). The quality of isolated RNA was checked on an agarose gel and quantified using the ultraviolet (UV) spectrophotometry. cDNA was synthesized from 2 μg of total RNA using PrimeScript™ RT Master Mix (Takara, RR036A). Real-time PCR was performed with SYBR Green detection. An ABI Prism 7300 sequence detection system (Applied Biosystems) was used for the PCR cycling reaction, real-time data collection, and analysis. GAPDH was selected as the reference gene. The relative transcript levels were quantified by the 2^-ΔΔCT^ method. The qRT-PCR primers are listed in **Supplementary Table 1**.

### Western Blot Analysis

Isolated Monocytes were lysed with RIPA lysis buffer (Beyotime Biotechnology, P0013B) containing protease inhibitors (Roche, 04693132001) and phosphatase inhibitors (Roche, 04906845001), and lysed on ice for 30 minutes. The lysate was clarified by centrifugation at 15000 g at 4°C for 15 minutes. Protein concentration was measured by the bicinchoninic acid assay (Beyotime Biotechnology, P0009). Separate equal amounts of protein from all samples by SDS-PAGE and transfer to PVDF membrane (Merck Millipore, ISEQ00010). The membranes were blocked with 5% (w/v) non-fat milk in PBS containing 0.1%(v/v) Tween-20 for 1 hour and incubated at 4°C overnight with primary antibodies, CD14 (1:1000; Abcam; ab221678), MyD88 (1:800; Abcam; ab219413), p65-NFκB (1:1000; Abcam; ab32536), p65-NFκB phospho S536 (1:1000; Abcam; ab76302), GAPDH (1:5000; Sigma-aldrich; G9545). Immunoreactive bands were detected by horseradish peroxidase–labeled secondary antibodies using SuperSignal™ West Pico Plus (ThermoFisher Scientific, 34577). Prestained protein ladders were used to estimate the molecular weights (ThermoFisher Scientific, 26616). Merge image and images of each protein hybridization membrane is shown in **Supplementary Figure 3**. Protein band intensity was measured using Image J software.

### Statistical Analysis

Statistical analysis was performed using GraphPad Prism 7 Software. To compare two groups, independent sample T-test was used for western blot and immunohistochemistry analysis of human tissue samples. To compare more than two groups, a one-way ANOVA test with Tukey’s multiple comparisons *post-hoc* test was used. Data are expressed as means ± SEM of triplicate samples drawn from a minimum of three independent experiments, and a value of P<0.05 was considered statistically significant.

## Results

### LPA_3_ Is Essential in the Fight Against Sepsis

**Figure 1:**
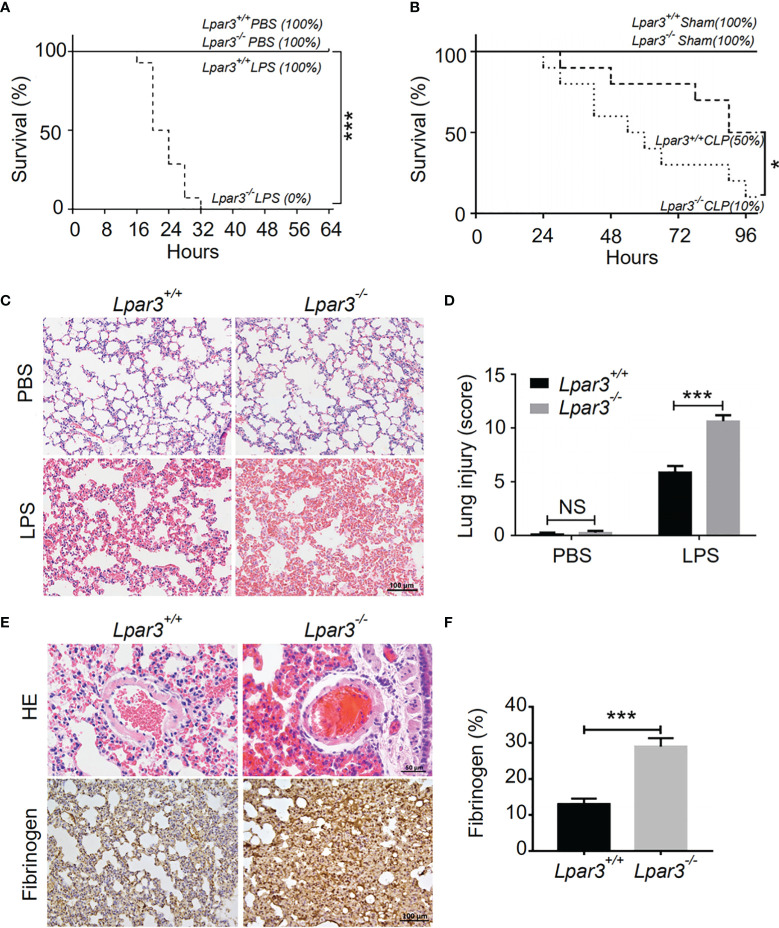
LPA_3_ deficiency reduce the survival rate of mice with sepsis, aggravated damage and microthrombus formation in the lungs of mice with sepsis. **(A)**: The survival rate of WT and *Lpar3^-/^* ^-^ mice under LPS-induced sepsis model. WT and *Lpar3^-/^* ^-^ mice intraperitoneally injected with PBS or LPS, monitored every 4 h for a 64-h period. *Lpar3^-/^* ^-^ mice showed lower survival rate than WT mice after LPS injection (n = 6 for *Lpar3^+/+^* or *Lpar3^-/-^* control, n = 16 for *Lpar3^+/+^* or *Lpar3^-/-^* LPS). **(B)**: The survival rate of WT and *Lpar3^-/^* ^-^ sepsis mice under CLP-induced sepsis model. WT and *Lpar3^-/^* ^-^ mice intraperitoneally injected with PBS or LPS, monitored every 6 h for a 96-h period. *Lpar3^-/^* ^-^ mice showed lower survival rate than WT mice after LPS injection (n = 6 for *Lpar3^+/+^* or *Lpar3^-/-^* control, n = 10 for *Lpar3^+/+^* or *Lpar3^-/-^* CLP). **(C, D)** H&E staining of the lungs of WT and *Lpar3^-/^* ^-^ mice. *Lpar3^-/^* ^-^ mice showed increased lung injury than WT mice after LPS injection, the individuals who graded the severity of lung injury were blinded to the genotype. (n = 8 for each group); Bar = 100 μm. **(E, F)** H&E staining of thrombus and immunohistochemical staining of fibrinogen in the lungs of WT or *Lpar3^-/^* ^-^ mice. Thrombus was found in small blood vessels of *Lpar3^-/^* ^-^ sepsis mouse lungs; Bar = 50 μm. Lung fibrinogen levels were higher in *Lpar3^-/^* ^-^ sepsis mice compared to WT controls (n = 8 for each group); Bar = 100 μm; *P < 0.05, *** P < 0.0001. NS, no significance. LPA_3_, lysophosphatidic acid receptor 3; WT, wild-type; PBS phosphate-buffered saline; LPS, lipopolysaccharide; H&E, hematoxylin and eosin.

To explore the potential role of LPA_3_ in sepsis, Lipopolysaccharide (LPS) or an equal volume of phosphate-buffered saline (PBS) as a control was injected intraperitoneally into wide-type (WT) and *Lpar3* ^-/-^ mice. The survival rate was monitored every 4 hours for 64 hours. When mice were administered 7 mg/kg LPS, all *Lpar3* ^-/-^ mice with sepsis died within 32 hours, with a survival rate of 0%. WT mice with sepsis continued to survive until the end of the test period (64 hours) with a 100% survival rate (**Figure 1A**). To further confirm this result, another sepsis model, cecal ligation and puncture (CLP) model were also made and survival rate was recorded every 6 hours for a 96 hours period. CLP mice also showed differences in survival rates, with 50% in the WT mice and only 10% in the *Lpar3* ^-/-^ mice to the end of the test period (96 hours) (**Figure 1B**). This result suggests that LPA_3_ deficiency significantly reduces the survival rate of mice with sepsis and that LPA_3_ plays an important role in the pathogenesis of sepsis, especially endotoxin-induced sepsis.

**Figure 2:**
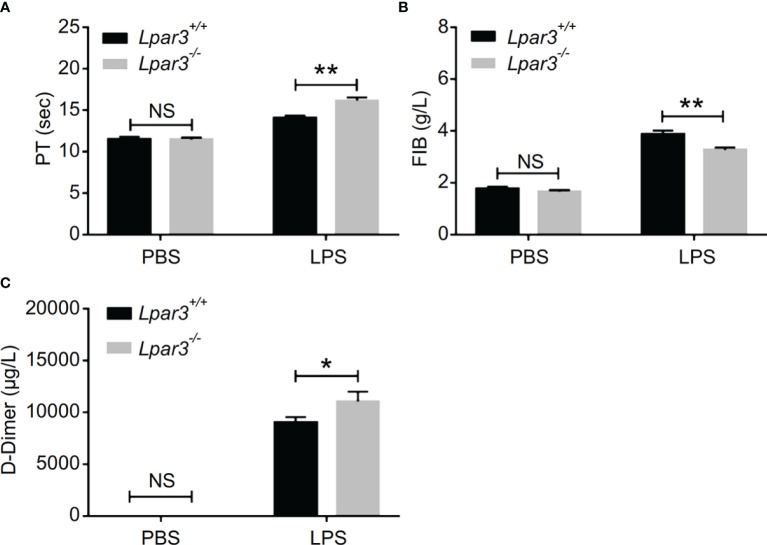
LPA_3_ deficiency exacerbates coagulation abnormalities and thrombosis in mice with sepsis. **(A–C)** The circulating levels of coagulation markers in the mice after LPS injection. *Lpar3^-/-^* septic mice lengthened the PT, contained less FIB and more D-dimers compared with WT septic mice (n = 8 for each group); *P < 0.05, **P < 0.01. NS, no significance. LPA_3_, lysophosphatidic acid receptor 3; WT, wild-type; LPS, lipopolysaccharide; PT, plasma prothrombin time; FIB, fibrinogen.

To further investigate organ damage in mice with sepsis, organs were collected 12 hours after LPS injection. Hematoxylin and eosin (H&E) staining showed significant hyperplasia of the splenic red marrow in *Lpar3* ^-/-^ mice with sepsis (**Supplementary Figure 1**), suggesting that LPA_3_ deficiency caused a more intense inflammatory response in sepsis. In addition to the spleen, H&E staining revealed increased levels of lung injury in *Lpar3* ^-/-^ mice with sepsis (**Figures 1C, D**), and microthrombi were present in the small vessels of the lungs of *Lpar3* ^-/-^ mice with sepsis (**Figure 1E**). To further verify this assumption, we measured fibrin/fibrinogen content in the lungs. Immunohistochemical staining revealed that LPA_3_ deficiency resulted in a significant increase in fibrin/fibrinogen content in the lungs of mice with sepsis (**Figures 1E, F**). Given this information, we hypothesized that LPA_3_ deficiency may cause DIC in mice with sepsis. During sepsis, the ongoing activation of coagulation pathways can overwhelm the anticoagulant systems and result in consumptive coagulopathy or DIC. Blood coagulation measurements demonstrated that, compared with that in WT mice with sepsis, *Lpar3* ^-/-^ mice with sepsis had significantly longer plasma prothrombin time (PT) (**Figure 2A**), less fibrinogen levels (FIB) (**Figure 2B**), and more D-dimer content (**Figure 2C**). This data seemed to confirm that the mice had experienced a severe coagulation event, resulting in excessive thrombin consumption. In addition, we observed that *Lpar3* ^-/-^ mice with sepsis had almost no epidermal blood flow (**Supplementary Figures 2A, B**), and microcirculatory perfusion was seriously blocked. These results suggest that LPA_3_ plays an important role in the maintenance of normal coagulation during sepsis.

### The Absence of LPA_3_ Induced Excessive NETs in Sepsis

**Figure 3:**
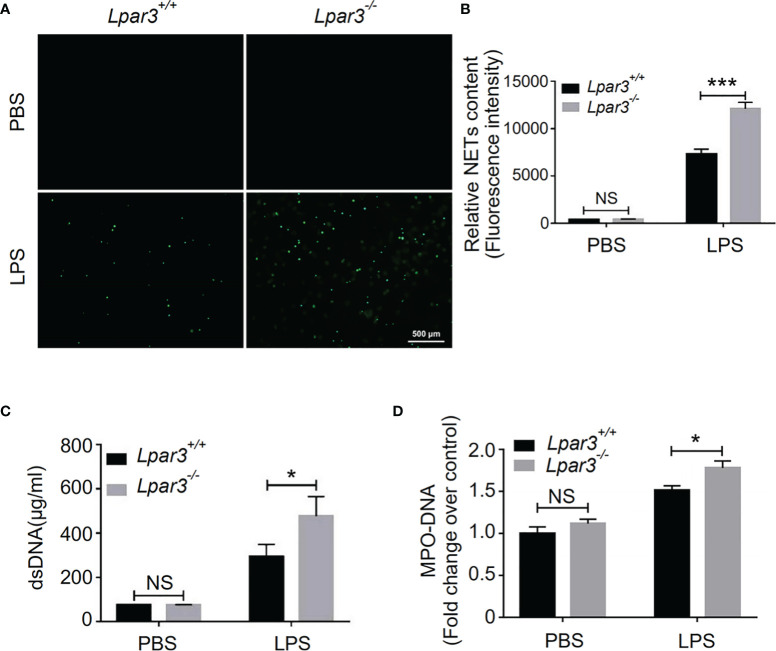
LPA_3_ deficiency induces excessive NETs in mice with sepsis. **(A, B)** Sytox-green staining of NETs induced by the plasma from septic mice. Plasma from *Lpar3^-/-^* septic mice induced more NETs than WT controls (n = 8 for each group); Bar = 500 μm. **(C)** The circulating level of dsDNA in the mice after LPS injection. The level of dsDNA was much higher in the plasma of *Lpar3^-/-^* septic mice than in that of WT controls (n = 6 for *Lpar3^+/+^* or *Lpar3^-/-^* control, n = 9 for *Lpar3^+/+^* LPS, n = 7 for *Lpar3^-/-^* LPS) **(D)** The circulating level of MPO-DNA complexes in the mice after LPS injection. The level of MPO-DNA complexes was much higher in the plasma of *Lpar3^-/-^* sepsis mice than in that of WT controls (n =10 for each group); *P < 0.05, ***P < 0.0001. NS, no significance. LPA_3_, lysophosphatidic acid receptor 3; WT, wild-type; PBS phosphate-buffered saline; LPS, lipopolysaccharide; H&E, hematoxylin and eosin; NETs, neutrophil extracellular traps.

NETs play an important role against infection in septic patients and animal models. However, there is growing evidence that excessive NETs formation during sepsis contributes to organ injury, intravascular thrombosis, and DIC (29, 32–35). Recently, we found that LPA regulates thrombotic stability *in vitro* by inducing the release of NETs from neutrophils (39). Therefore, we hypothesized that LPA receptors might be involved in the regulation of NETs release. To confirm that LPA_3_ affects the production of NETs during sepsis, we isolated plasma from WT and *Lpar3* ^-/-^ mice with sepsis, co-cultured with isolated WT neutrophils separately. The results showed that the plasma of *Lpar3* ^-/-^ mice with sepsis had an enhanced ability to induce NETs compared to that of the WT controls (**Figures 3A, B**). Ds-DNA and MPO-DNA complexes are major indicators of NETs in plasma, detection of dsDNA and MPO-DNA levels in the plasma revealed its increased levels in *Lpar3* ^-/-^ mice when compared to that in the WT controls after LPS injection (**Figures 3C, D**). We also performed immunofluorescence assays for NETs markers in lung tissue and found that the expression of CitH_3_ and MPO were much higher in *Lpar3* ^-/-^ mice than in WT controls after LPS injection (**Supplementary Figures 2C–F**). These results imply LPA_3_ is associated with the release of NETs during sepsis.

**Figure 4:**
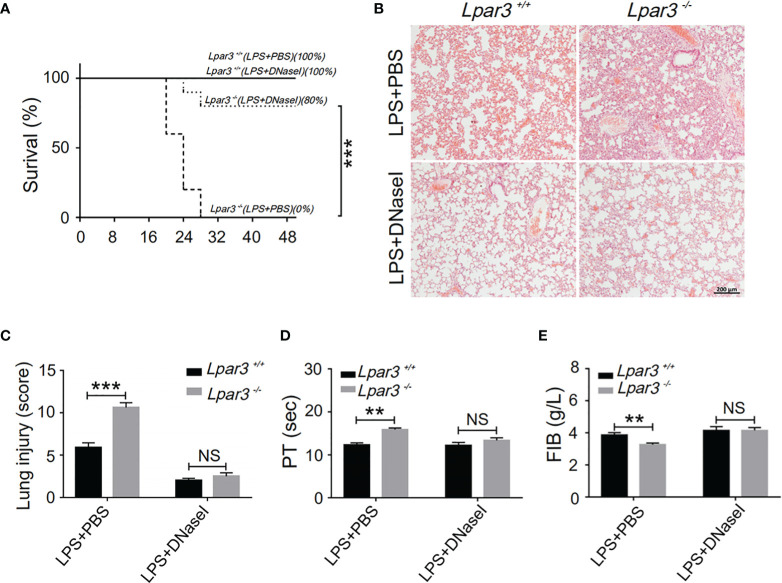
NETs scavenger DNaseI reduces organ damage and coagulation irregularities in sepsis **(A),** The survival rate of septic mice with DNaseI. WT and *Lpar3^-/^* ^-^ septic mice intravenously injected with PBS or DNaseI, monitored every 4 h for a 48-h period, and DNaseI-treated *Lpar3^-/-^* septic mice showed a higher survival rate than PBS-treated *Lpar3^-/-^* septic mice (n = 10 for each group); **(B, C),** H&E staining of the lungs of PBS-treated or DNaseI-treated mice. DNaseI-treated *Lpar3^-/-^* septic mice showed less lung damage compared with PBS-injected *Lpar3^-/-^* controls; the individuals who graded the severity of lung injury were blinded to the genotype (n = 8 for each group). **(D, E),** The circulating levels of coagulation markers PT and FIB in the PBS-treated or DNaseI-treated septic mice (n = 5 for each group). **P < 0.01, ***P < 0.0001. NS, no significance. LPA_3_, lysophosphatidic acid receptor 3; WT, wild-type; PBS phosphate-buffered saline; LPS, lipopolysaccharide; H&E, hematoxylin and eosin; NETs, neutrophil extracellular traps; DNase I, deoxyribonuclease I.

One of the major components of NETs are extracellular DNA networks, making DNaseI an effective NETs scavenger. To explore the role of NETs in the development of sepsis in *Lpar3* ^-/-^ mice, DNaseI (or PBS alone as a control) was injected into LPS-induced septic mice through the tail vein. DNaseI-treated *Lpar3* ^-/-^ mice with sepsis exhibited significantly higher survival rates than the PBS-treated *Lpar3* ^-/-^ mice with sepsis (**Figure 4A**). Similarly, the score of lung injury was significantly reduced compared to that in the PBS-injected *Lpar3* ^-/-^ mice (**Figures 4B, C**). Moreover, there was no difference between *Lpar3* ^-/-^and WT mice with sepsis in lung injury, PT and FIB after DNaseI treatment (**Figures 4C–E**). Together, these results showed that excessive NETs generation in *Lpar3* ^-/-^ sepsis mice may be the main cause of their aggravated sepsis injury.

### LPA_3_ Deficiency Affects Plasma Levels of NETs-Inducers Interleukin (IL)-6 and IL-8

**Figure 5:**
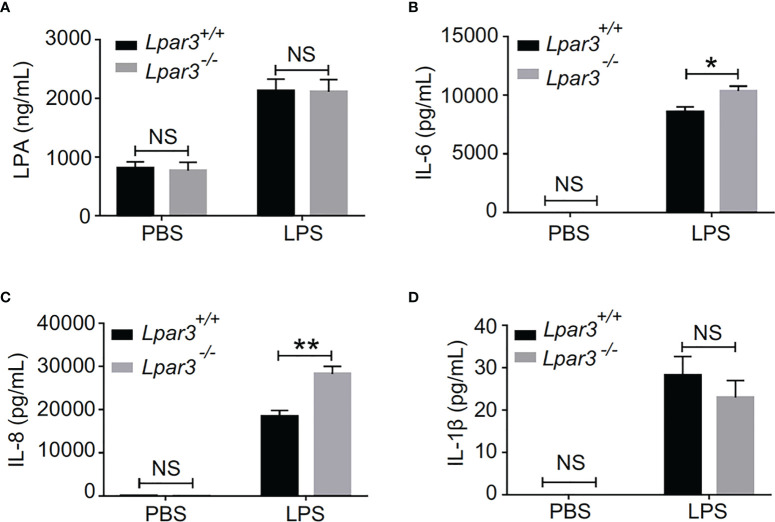
LPA_3_ deficiency affects the level of IL-6 and IL-8 in the plasma of mice with sepsis. **(A),** Plasma LPA levels were significantly increased in LPS-treated mice; however, LPA_3_ deficiency did not significantly affect plasma LPA levels in sepsis mice. (n = 12 for each group); **(B, C):** The plasma levels of the cytokines IL-6, and IL-8 were determined after sepsis induction by LPS, IL-6 and IL-8 in the plasma revealed higher levels in *Lpar3^-/-^* mice with sepsis. (n = 8 for *Lpar3^+/+^* or *Lpar3^-/-^* control, n = 9 for *Lpar3^+/+^* LPS, n = 10 for *Lpar3^-/-^* LPS). **(D),** Plasma IL-1β levels were no difference in LPS-treated WT and *Lpar3* ^-/-^ mice. (n = 9 for *Lpar3^+/+^* or *Lpar3^-/-^* controls, n = 8 for *Lpar3^+/+^* LPS, n =6 for *Lpar3^-/-^* LPS); *P < 0.05, **P < 0.01. NS, no significance. LPA_3_, lysophosphatidic acid receptor 3; LPS, lipopolysaccharide; IL-6, interleukin-6; IL-8, interleukin-8.

The plasma of *Lpar3* ^-/-^ mice with sepsis is more capable of inducing NETs, suggesting the presence of more inducers. Recently, we identified LPA as an inducer of NETs (39). Therefore, we considered whether LPA_3_ deficiency would cause changes in the content of LPA and thus induce the production of excess NETs. We measured plasma LPA levels by enzyme-linked immunoassay (ELISA) and found that plasma LPA levels were significantly increased in septic mice, but LPA_3_ deficiency did not significantly affect plasma LPA levels in mice with sepsis (**Figure 5A**). As previously mentioned, LPA_3_ deficiency caused an increased inflammatory response in sepsis (**Supplementary Figure 1**); so, we examined the expression of IL-1β, IL-6 and IL-8, which are the major pro-inflammatory cytokines reported to be capable of directly inducing NETs in mouse plasma. The results showed that the plasma levels of IL-6 and IL-8 were higher in *Lpar3* ^-/-^ sepsis mice than in WT mice (**Figures 5B, C**), while IL-1β had no difference (**Figure 5D**). Excess IL-6 and IL-8 could stimulate neutrophils and increase NETs levels. Therefore, these results suggest plasma IL-6 and IL-8, but not plasma LPA and IL-1β, are responsible for the excessive release of NETs in *Lpar3* ^-/-^ mice with sepsis.

### LPA_3_ Acts on Monocytes in Sepsis

**Figure 6:**
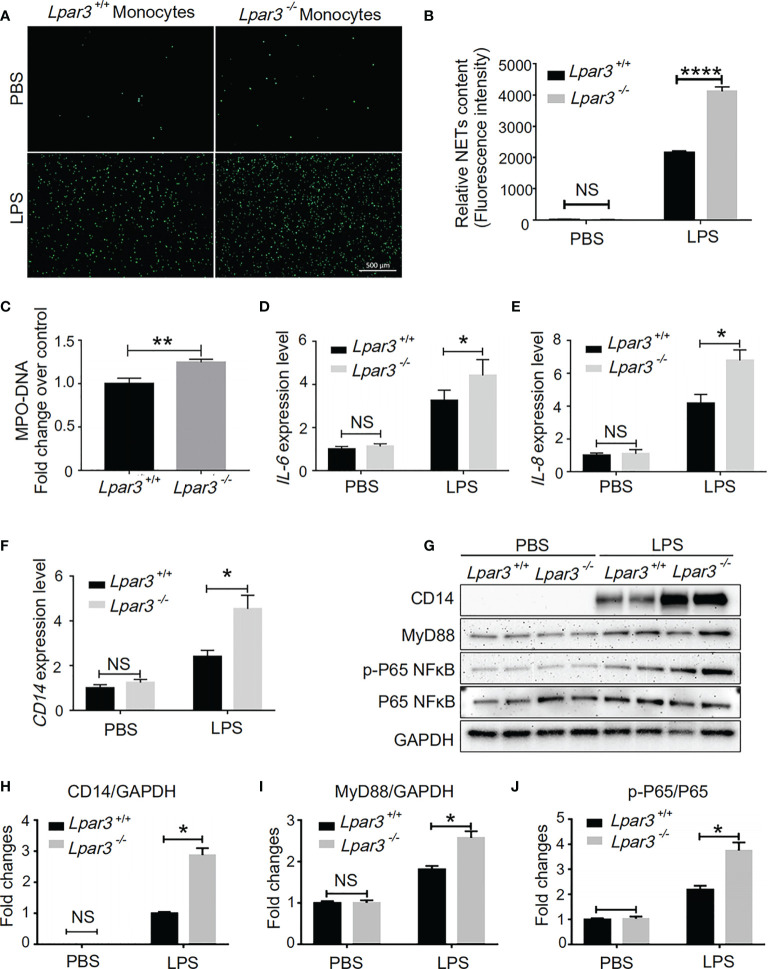
LPA_3_ works on monocytes in sepsis. **(A, B),** SYTOX Green staining of NETs induced by WT or *Lpar3^-/-^* monocytes with LPS. Neutrophils co-cultured with *Lpar3^-/-^* monocytes were induced more NETs after LPS stimulation than neutrophils co-cultured with WT monocytes (n = 6 for each group), Bar = 100 μm. **(C),** The level of MPO-DNA complexes in the cell culture supernatant after LPS treatment. Neutrophils co-cultured with *Lpar3^-/-^* monocytes had higher levels of the MPO-DNA in the cell culture supernatant than in that of WT controls (n =9 for each group). **(D–F),** qRT-PCR analysis of gene expression in monocytes. LPA_3_ deficiency increased the expression of IL-6, IL-8, and CD14 in monocytes from mice with sepsis (n = 6 for each group). **(H–J),** Western blot analysis of protein levels in monocytes. The expression of monocyte protein CD14, MyD88, and p-P65 NFκB increased in *Lpar3^-/-^* mice with sepsis (n = 6 for each group). *P < 0.05, **P < 0.01, ****P < 0.00001. NS, no significance. LPA_3_, lysophosphatidic acid receptor 3; WT, wild-type; LPS, lipopolysaccharide; NETs, neutrophil extracellular traps.

Monocytes are one of the main sources of IL-6 and IL-8. Thus, we speculate LPA_3_ from monocytes plays a role in the development of sepsis. We co-cultured WT mouse neutrophils with monocytes from *Lpar3* ^-/-^ or WT mice under stimulation with LPS or PBS control. SYTOX-Green staining showed that neutrophils co-cultured with *Lpar3* ^-/-^ monocytes were more likely to release NETs after LPS treatment (**Figures 6A, B**), and also had higher levels of the MPO-DNA in the cell culture supernatant (**Figure 6C**). These results indicated that LPA_3_ in monocytes resists the release of NETs during sepsis.

We investigated the effect of LPA_3_ on expression of IL-6 and IL-8 in monocytes. We found that LPS treatment increased the RNA expression levels of IL-6 and IL-8 in monocytes isolated from septic model, whereas LPA_3_ deficiency further increased the expression levels of IL-6 and IL-8 in monocytes (**Figures 6D, E**). This result indicates that monocyte LPA_3_ plays an important role in the elevation of plasma IL-6 and IL-8 levels in LPS-induced *Lpar3* ^-/-^ mice with sepsis.

In monocytes, LPA was found to enhance the ability of LPS to induce CD14 expression (43). For this reason, we investigated whether LPA_3_ deficiency affected the level of CD14 on the surface of monocytes in mice with sepsis. qRT-PCR and western blotting revealed that CD14 expression was significantly increased in monocytes from *Lpar3* ^-/-^ mice with sepsis (**Figures 6F–H**). As an LPS receptor, increased expression of CD14 leads to further amplification of LPS signaling.

CD14 binds to Toll-like receptor 4 (TLR4) on the monocyte cytosolic membrane and further activates downstream signaling pathways, such as NFκB and MAPK through the MyD88-dependent pathway or TRIF-dependent pathway, exerting a wide range of biological effects. To elucidate the changes in the molecular mechanisms of monocytes in *Lpar3^-/-^* mice with sepsis, we examined the related signaling pathway proteins. The results revealed that monocyte MyD88 protein and p-P65 NFκB protein expression were significantly increased in *Lpar3* ^-/-^ mice with sepsis (**Figures 6I, J**). These results indicate LPA_3_ deficiency may induce excess NETs through the MyD88-NFκB signaling pathway.

### LPA_3_ Agonist (2S)-(OMPT) as a Potential Therapeutic Strategy for Sepsis

**Figure 7:**
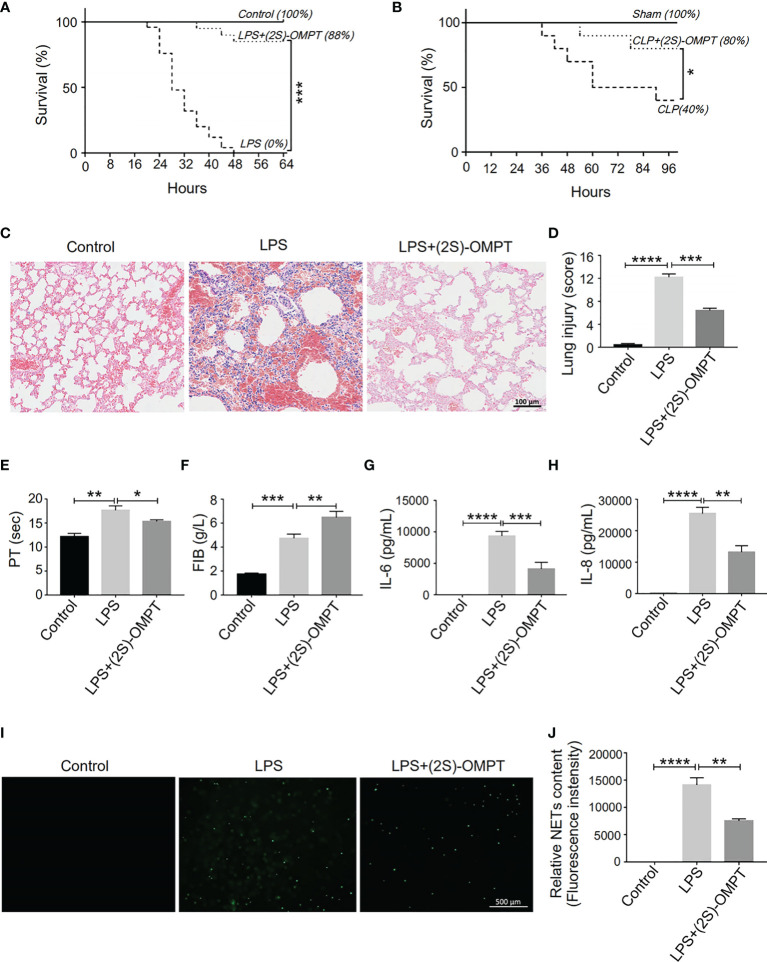
LPA_3_ agonist (2S)-OMPT possess potential therapeutic effect for sepsis. **(A),** The survival rate of LPS-induced septic mice with (2S)-OMPT. Mice were treated with (2S)-OMPT after LPS injection, monitored every 4 h for a 64-h period. (2S)-OMPT treatment improved the survival rate of mice with sepsis (control n = 6, LPS n = 16, LPS+(2S)-OMPT n = 16). **(B),** The survival rate of CLP-induced sepsis mice with (2S)-OMPT. Mice were treated with (2S)-OMPT after CLP, monitored every 6 h for a 96-h period. (Control n = 6, CLP n = 10, CLP+(2S)-OMPT n = 10) **(C, D),** H&E staining of the lungs of mice with sepsis with (2S)-OMPT. (2S)-OMPT treatment decreased lung injury of mice with sepsis (n = 5), Bar =100 μm. **(E, F),** The circulating levels of coagulation markers in the mice with sepsis with (2S)-OMPT. (2S)-OMPT treatment shortened PT, increased FIB levels, reduced the risk of thrombosis (control n = 6, LPS n = 7, LPS+(2S)-OMPT n = 7). **(G, H),** The plasma levels of the cytokines IL-6 and IL-8 in the mice with sepsis with (2S)-OMPT. (2S)-OMPT injection decreased the plasma content of IL-6 and IL-8 (n = 10). **(I, J),** SYTOX Green staining of NETs induced by the plasma from septic mice with (2S)-OMPT, (2S)-OMPT treatment decreased NETs formation of mice with sepsis (n = 8). *P < 0.05, **P < 0.01, ***P < 0.0001, ****P < 0.00001. LPA_3_, lysophosphatidic acid receptor 3; LPS, lipopolysaccharide; (2S)-OMPT; (2S)-1-oleoyl-2-methylglycero-3-phosphothionate; BSA, bovine serum albumin; PT, plasma prothrombin time; FIB, fibrinogen; NETs, neutrophil extracellular traps.

The absence of LPA_3_ induced excessive NETs and caused increased organ damage and more thrombosis. Therefore, we hypothesized that (2S)-OMPT, an LPA_3_ selective agonist, could have therapeutic implications for sepsis and sepsis-related thrombosis. We constructed LPS-induced and CLP-induced sepsis model in WT mice and injected (2S)-OMPT into septic mice *via* tail vein 1 hour after LPS injection or CLP to investigate its therapeutic effect on sepsis. Specifically, in the LPS-induced model, to reflect the difference in mortality in WT mice, we increased the LPS dose to 15 mg/kg. (2S)-OMPT injection significantly improved the survival rate in both LPS-induced and CLP-induced sepsis (**Figures 7A, B**) and decreased lung injury in LPS-induced septic mice compared with that in the controls (**Figures 7C, D**). Furthermore, (2S)-OMPT injection shortened the PT, increased the FIB content (**Figures 7E, F**), and reduced the degree of microcirculatory prefusion embolism (**Supplementary Figure 4**) in mice with sepsis. This significantly reduced the risk of abnormal coagulation and sepsis-related thrombosis. As previously mentioned, aggravated septic injury is related to LPA_3_ due to excessive NETs induced by increased levels of IL-6 and IL-8. (2S)-OMPT injection decreased the levels of plasma IL-6 and IL-8 (**Figures 7G, H**) and reduced the formation of NETs (**Figures 7I, J**). These results demonstrate (2S)-OMPT has therapeutic implications for sepsis-related tissue damage and thrombosis through inhibition of IL-6 and IL-8 induced NETs formation, possibly contributing to the future clinical treatment of sepsis.

## Discussion

**Figure 8:**
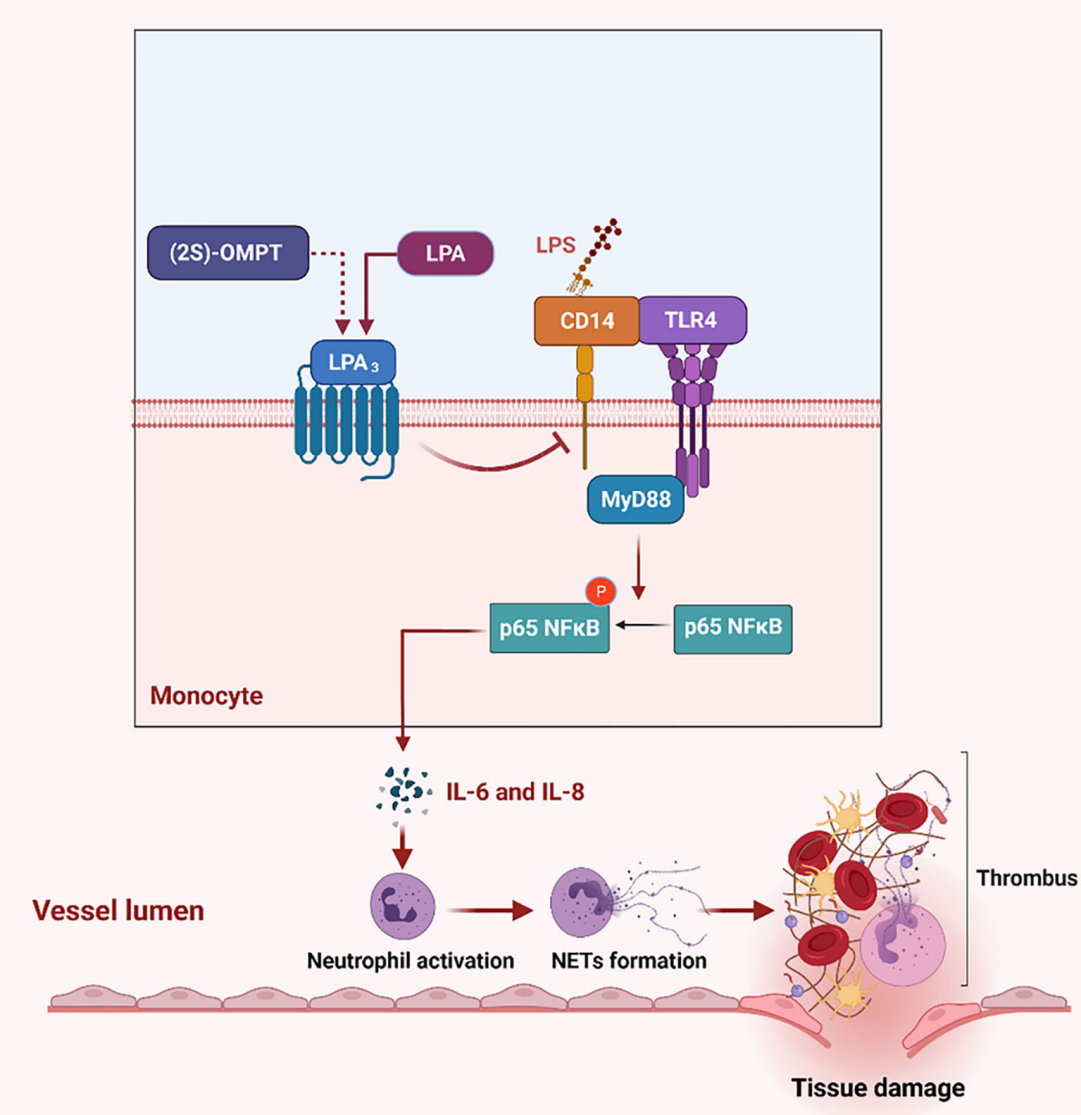
The role of LPA_3_ protects against the organism by affecting the TLR4 signaling pathway and NETs release. During sepsis, LPA_3_ deficiency monocytes are stimulated to express more quantities of CD14, which interacts with LPS and activates TLR4 signaling pathway. Activation of the TLR4-MyD88-p65 NFκB signaling pathway controls inflammatory cytokines (IL-6 and IL-8) release, induces excessive NETs, further causes thrombosis and tissue damage. (2S)-OMPT, the agonist of LPA_3_, inhibits the inflammatory cytokines and NETs formation, reduces tissue damage and septic lethality, to be a therapeutic improvement in sepsis treatment. Illustration created with BioRender. Abbreviations: LPA_3_, lysophosphatidic acid receptor 3; NETs, neutrophil extracellular traps; LPS, lipopolysaccharide; TLR4, Toll-like receptor 4. (2S)-OMPT; (2S)-1-oleoyl-2-methylglycero-3-phosphothionate.

Using the sepsis model and genetic mice, we identified a major protective role for LPA_3_ in sepsis through the inhibition of NETs generation and NETs related thrombosis. Activation of LPA_3_ downregulated CD14 expression on monocytes and inhibited the TLR4 signaling pathway, further leading to a decrease in the levels of NETs-inducers IL-6 and IL-8. In addition to lessening the formation of NETs, sepsis-related tissue damage and thrombosis were reduced (**Figure 8**). Therefore, the survival rate was significantly improved after LPA_3_ agonist injection. By exploring the role and mechanism of LPA signaling in the pathophysiology of sepsis, our study may provide a novel mechanism for the occurrence and development of sepsis and provide an innovative therapeutic strategy.

NETs have attracted much attention in the scope of sepsis because of their close relationship with infection and thrombosis. In this study, we found that the levels of NETs in *Lpar3^-/-^* mice were higher compared to that in WT mice after LPS injection. The destruction of NETs by DNase I treatment significantly reduced lung injury and mortality in LPS-induced *Lpar3^-/-^* mice. Plasma from *Lpar3* ^-/-^ mice with sepsis was found to have an enhanced ability to induce NETs compared to that from the WT controls, which suggests that the plasma of *Lpar3* ^-/-^ mice with sepsis contain more NETs inducers. The protective role of LPA_3_ agonists in sepsis and regulatory effects in NETs production were confirmed, which may be a new strategy for the treatment of sepsis.

LPA is a small ubiquitous lipid found in vertebrate and nonvertebrate organisms that mediates diverse biological actions and demonstrates medicinal relevance. LPA can exert a wide range of biological effects by binding to the LPA receptors (LPA_1-6_) and signal through numerous effector pathways activated by heterotrimeric G proteins, including G_i/o_, G_12/13_, G_q_, and G_s_ (44). Disturbances in normal LPA signaling may contribute to a range of diseases, including neuropsychiatric disorders, asthma, acute lung injury, fibrosis, and cardiovascular disease (36–38, 45–47). LPA_3_ couples to G_αi/o_ and G_αq/11_ and mediates LPA-induced Ca^2+^ mobilization, inhibition and activation of adenylate cyclase, activation of PLC, and activation of MAPK (48). *Lpar3^-/-^* female mice have delayed embryo implantation, embryo crowding, and reduced litter size (41). LPA_3_ are also expressed in the testis (49, 50), suggesting a role for LPA_3_ signaling in male sperm behavior and fertility.

LPA level was elevated in the plasma of sepsis patients and LPS-induced animal models (51, 52). The contribution of LPA in stimulating the NETs release *in vitro* was found by our group (40). However, in this study we found that LPA_3_ deficiency did not affect the plasma LPA content. Therefore, LPA_3_ affects the generation of NETs in other ways. One of most important findings in this study concerns the increased levels of plasma IL-6 and IL-8 in *Lpar3^-/-^* mice. Monocytes are the main source of cytokine synthesis. We showed that LPA_3_ deficiency upregulated CD14 expression on monocytes and overactivated the MyD88-NFκB signaling pathway, resulting in excessive release of IL-6 and IL-8. LPA_3_ can regulate IL-6/IL-8 release, reduce the formation of NETs and thrombosis, and improve the survival rate of mice with sepsis effectively. Regulating inflammatory factors to block NETs production would be a novel direction of sepsis treatment.

Notably, most of the studies in the field of LPA and inflammation focus on LPA_1_, and support the role of LPA_1_ in promoting LPS-induced inflammatory responses (39, 53). CD14 expression in monocytes was elevated after LPA_1_ activation (43) and interact with LPA_1_ in response to LPS treatment in lung injury (39, 53, 54). However, the exploration of biological properties and mechanisms of LPA_3_ had not been investigated. Our group found a balanced regulation between LPA_1_ and LPA_3_ in cardiomyocytes through microRNAs (55). LPA promotes *miR-23a* expression through LPA_3_, and *miR-23a* downregulates the expression of LPA_1_. In this study, we found that LPA_3_ deficiency promoted the LPS-induced elevation of CD14 expression, caused an up-regulation of TLR4 signaling in monocytes, and aggravated sepsis injury. However, the mutual regulation of LPA_1_ and LPA_3_ and its relevant mechanisms in sepsis requires further investigation.

Although one of the leading causes of sepsis is bacterial infection, complications caused by other infection routes can also lead to sepsis. For example, viral infections caused by SARS-CoV-2 can ultimately cause sepsis and end-organ dysfunction. A recent study found that LPA_1_ colocalizes with the SARS-CoV-2 receptor ACE2. Inhibition of LPA_1_ could enhance IFN-I/III levels and antiviral responses (56). This study strongly demonstrates the role of LPA receptors in both bacterial and viral infections, and poses LPA_1_ and LPA_3_ as potential drug targets for the treatment of multiple infections.

Many clinical trials have been conducted worldwide to evaluate the efficacy of different treatments for sepsis. However, none of these treatments have been successful in improving the high morbidity and mortality rates related to sepsis. Therefore, treatment of sepsis remains one of the most important challenges in global health. Inhibition of LPA_1_ with the antagonist ki16425 attenuated LPS-induced inflammation (39), making ki16425 a potential drug targeting LPA receptors. What needs to be emphasized here is that ki16425 inhibited both LPA_1_ and LPA_3_, but according to our findings, LPA_3_ plays protective functions in sepsis. Therefore, it would be wise to choose a more specific LPA receptor agonists or antagonists for the treatment of sepsis. As a specific agonist of the LPA_3_ receptor, (2S)-OMPT was found to reduce the inflammatory response, decrease the production of NETs, reduce organ damage, and improve coagulation function. This effectively improved the survival rate of mice with sepsis in our study. Consequently, we believe (2S)-OMPT has excellent drug translation and application prospects. However, our study used (2S)-OMPT in early sepsis. Based on previous sepsis studies, there is an a 7% increased risk of death for each hour anti-bacterial treatment is delayed (57). Therefore, a timely initiation of drug therapy in suspected sepsis is associated with improved patient outcomes.

Diverse animal models have been developed to study the pathophysiology of sepsis. Administration of exogenous toxins, such as LPS, and cecum ligation and puncture (CLP) are the most commonly used. In our study, we used both the LPS-induced and the CLP-induced sepsis models to explore the survival rate of LPA_3_ mice. It was found that although WT mice exhibited higher survival rates than LPA_3_ deficiency mice in both models, the difference was more pronounced in the LPS-induced model. This is mainly because of the difficulty of CLP-induced sepsis in controlling the severity of sepsis in practice (58). Our research focused on the impact of LPA_3_ in sepsis, which requires a better consistency. Therefore, we chose the LPS-induced sepsis model, which is considered a more highly controlled and standardized model (58). However, CLP-induced sepsis was better to simulate clinical symptoms, this makes our animal model not completely consistent with the development of clinical sepsis, remains one of the problems that need to be solved in the future studies.

In conclusion, we identified LPA_3_ as a critical suppressor of sepsis through the regulation of both NETs production and thrombosis. Targeting LPA_3_ could represent a novel therapeutic approach to limit sepsis or other severe inflammatory disease. Expansion to human subjects should be evaluated in the future.

## Data Availability Statement

The datasets presented in this study can be found in online repositories. The names of the repository/repositories and accession number(s) can be found below: https://figshare.com/, 10.6084/m9.figshare.17696591.

## Ethics Statement

The animal study was reviewed and approved by The Laboratory Animal Management and Use Committee of Fuwai Hospital, Chinese Academy of Medical Sciences.

## Author Contributions

SP, CX, FW, and XC conceived and designed the research. SP and CX performed statistical analysis. XC and FW handled funding and supervision. SP, CX, FW, JP, RB, RP, and TL acquired the data. JC provided gene deficiency mice. Advice for the project was received from XFC and JZ. SP drafted the manuscript. FW and XC made critical revision of the manuscript for key intellectual content. All authors contributed to the article and approved the submitted version.

## Funding

This work was supported by grants from the Natural Science Foundation of China (81770304) and (82172334), and the CAMS Innovation Fund for Medical Sciences (CIFMS) (2017-I2M-3-003).

## Conflict of Interest

The authors declare that the research was conducted in the absence of any commercial or financial relationships that could be construed as a potential conflict of interest.

## Publisher’s Note

All claims expressed in this article are solely those of the authors and do not necessarily represent those of their affiliated organizations, or those of the publisher, the editors and the reviewers. Any product that may be evaluated in this article, or claim that may be made by its manufacturer, is not guaranteed or endorsed by the publisher.

## Abbreviations

LPA, Lysophosphatidic acid; LPA_1,_ Lysophosphatidic acid receptor 1 (protein); LPA_3_, Lysophosphatidic acid receptor 3 (protein); *Lpar3*, Lysophosphatidic acid receptor 3 (gene); LPS, Lipopolysaccharide; NETs, Neutrophil extracellular traps; (2S)-OMPT, (2S)-1-(9Z-octadecenoyl)-2-O-methyl-glycero-3-phosphothionate.

## References

[1] Babyak JM Sharp CR. Epidemiology of Systemic Inflammatory Response Syndrome and Sepsis in Cats Hospitalized in a Veterinary Teaching Hospital. J Am Vet Med Assoc (2016) 249(1):65–71. doi: 10.2460/javma.249.1.65 27308883

[2] Levy MM Rhodes A Phillips GS Townsend SR Schorr CA Beale R. Surviving Sepsis Campaign: Association Between Performance Metrics and Outcomes in a 7.5-Year Study. Intensive Care Med (2014) 40(11):1623–33. doi: 10.1007/s00134-014-3496-0 25270221

[3] Singer M Deutschman CS Seymour CW Shankar-Hari M Annane D Bauer M. The Third International Consensus Definitions for Sepsis and Septic Shock (Sepsis-3). Jama (2016) 315(8):801–10. doi: 10.1001/jama.2016.0287 PMC4968574 26903338

[4] Hernandez G Bruhn A Ince C. Microcirculation in Sepsis: New Perspectives. Curr Vasc Pharmacol (2013) 11(2):161–9. doi: 10.2174/1570161111311020006 23506495

[5] Levi M van der Poll T Schultz M. New Insights Into Pathways That Determine the Link Between Infection and Thrombosis. Netherlands J Med (2012) 70(3):114–20. 22516575

[6] Ulloa L Brunner M Ramos L Deitch EA. Scientific and Clinical Challenges in Sepsis. Curr Pharm Des (2009) 15(16):1918–35. doi: 10.2174/138161209788453248 PMC3098530 19519432

[7] Dellinger RP Levy MM Carlet JM Bion J Parker MM Jaeschke R. Surviving Sepsis Campaign: International Guidelines for Management of Severe Sepsis and Septic Shock: 2008. Intensive Care Med (2008) 34(1):17–60. doi: 10.1007/s00134-007-0934-2 18058085 PMC2249616

[8] Castanheira FVS Kubes P. Neutrophils and NETs in Modulating Acute and Chronic Inflammation. Blood (2019) 133(20):2178–85. doi: 10.1182/blood-2018-11-844530 30898862

[9] Wang J. Neutrophils in Tissue Injury and Repair. Cell Tissue Res (2018) 371(3):531–9. doi: 10.1007/s00441-017-2785-7 PMC5820392 29383445

[10] Brinkmann V Reichard U Goosmann C Fauler B Uhlemann Y Weiss DS. Neutrophil Extracellular Traps Kill Bacteria. Sci (New York NY) (2004) 303(5663):1532–5. doi: 10.1126/science.1092385 15001782

[11] Byrd AS O'Brien XM Johnson CM Lavigne LM Reichner JS. An Extracellular Matrix-Based Mechanism of Rapid Neutrophil Extracellular Trap Formation in Response to Candida Albicans. J Immunol (Baltimore Md 1950) (2013) 190(8):4136–48. doi: 10.4049/jimmunol.1202671 PMC3622194 23509360

[12] Liu S Su X Pan P Zhang L Hu Y Tan H. Neutrophil Extracellular Traps are Indirectly Triggered by Lipopolysaccharide and Contribute to Acute Lung Injury. Sci Rep (2016) 6:37252. doi: 10.1038/srep37252 27849031 PMC5110961

[13] Muraro SP De Souza GF Gallo SW Da Silva BK De Oliveira SD Vinolo MAR. Respiratory Syncytial Virus Induces the Classical ROS-Dependent NETosis Through PAD-4 and Necroptosis Pathways Activation. Sci Rep (2018) 8(1):14166. doi: 10.1038/s41598-018-32576-y 30242250 PMC6154957

[14] Pilsczek FH Salina D Poon KK Fahey C Yipp BG Sibley CD. A Novel Mechanism of Rapid Nuclear Neutrophil Extracellular Trap Formation in Response to Staphylococcus Aureus. J Immunol (Baltimore Md 1950) (2010) 185(12):7413–25. doi: 10.4049/jimmunol.1000675 21098229

[15] Tanaka K Toiyama Y Inoue Y Araki T Mohri Y Mizoguchi A. Imaging Neutrophil Extracellular Traps in the Alveolar Space and Pulmonary Capillaries of a Murine Sepsis Model by Multiphoton Microscopy. Am J Respir Crit Care Med (2015) 191(9):1088–9. doi: 10.1164/rccm.201501-0121LE 25932768

[16] Etulain J Martinod K Wong SL Cifuni SM Schattner M Wagner DD. P-Selectin Promotes Neutrophil Extracellular Trap Formation in Mice. Blood (2015) 126(2):242–6. doi: 10.1182/blood-2015-01-624023 PMC4497964 25979951

[17] Joshi MB Lad A Bharath Prasad AS Balakrishnan A Ramachandra L Satyamoorthy K. High Glucose Modulates IL-6 Mediated Immune Homeostasis Through Impeding Neutrophil Extracellular Trap Formation. FEBS Lett (2013) 587(14):2241–6. doi: 10.1016/j.febslet.2013.05.053 23735697

[18] Mitroulis I Kambas K Chrysanthopoulou A Skendros P Apostolidou E Kourtzelis I. Neutrophil Extracellular Trap Formation is Associated With IL-1beta and Autophagy-Related Signaling in Gout. PloS One (2011) 6(12):e29318. doi: 10.1371/journal.pone.0029318 22195044 PMC3241704

[19] Yamada M Gomez JC Chugh PE Lowell CA Dinauer MC Dittmer DP. Interferon-Gamma Production by Neutrophils During Bacterial Pneumonia in Mice. Am J Respir Crit Care Med (2011) 183(10):1391–401. doi: 10.1164/rccm.201004-0592OC PMC3114063 21169470

[20] Koupenova M Corkrey HA Vitseva O Manni G Pang CJ Clancy L. The Role of Platelets in Mediating a Response to Human Influenza Infection. Nat Commun (2019) 10(1):1780. doi: 10.1038/s41467-019-09607-x 30992428 PMC6467905

[21] Yousefi S Mihalache C Kozlowski E Schmid I Simon HU. Viable Neutrophils Release Mitochondrial DNA to Form Neutrophil Extracellular Traps. Cell Death differ (2009) 16(11):1438–44. doi: 10.1038/cdd.2009.96 19609275

[22] Sayah DM Mallavia B Liu F Ortiz-Munoz G Caudrillier A DerHovanessian A. Neutrophil Extracellular Traps are Pathogenic in Primary Graft Dysfunction After Lung Transplantation. Am J Respir Crit Care Med (2015) 191(4):455–63. doi: 10.1164/rccm.201406-1086OC PMC4351593 25485813

[23] Clark SR Ma AC Tavener SA McDonald B Goodarzi Z Kelly MM. Platelet TLR4 Activates Neutrophil Extracellular Traps to Ensnare Bacteria in Septic Blood. Nat Med (2007) 13(4):463–9. doi: 10.1038/nm1565 17384648

[24] Caudrillier A Kessenbrock K Gilliss BM Nguyen JX Marques MB Monestier M. Platelets Induce Neutrophil Extracellular Traps in Transfusion-Related Acute Lung Injury. J Clin Invest (2012) 122(7):2661–71. doi: 10.1172/jci61303 PMC3386815 22684106

[25] McDonald B Urrutia R Yipp BG Jenne CN Kubes P. Intravascular Neutrophil Extracellular Traps Capture Bacteria From the Bloodstream During Sepsis. Cell Host Microbe (2012) 12(3):324–33. doi: 10.1016/j.chom.2012.06.011 22980329

[26] Gould TJ Vu TT Swystun LL Dwivedi DJ Mai SHC Weitz JI. Neutrophil Extracellular Traps Promote Thrombin Generation Through Platelet-Dependent and Platelet-Independent Mechanisms. Arterioscler Thromb Vasc Biol (2014) 34(9):1977–84. doi: 10.1161/ATVBAHA.114.304114 25012129

[27] Semeraro F Ammollo CT Morrissey JH Dale GL Friese P Esmon NL. Extracellular Histones Promote Thrombin Generation Through Platelet-Dependent Mechanisms: Involvement of Platelet TLR2 and TLR4. Blood (2011) 118(7):1952–61. doi: 10.1182/blood-2011-03-343061 PMC3158722 21673343

[28] Ammollo CT Semeraro F Xu J Esmon NL Esmon CT. Extracellular Histones Increase Plasma Thrombin Generation by Impairing Thrombomodulin-Dependent Protein C Activation. J Thromb Haemost (2011) 9(9):1795–803. doi: 10.1111/j.1538-7836.2011.04422.x 21711444

[29] Tanaka K Koike Y Shimura T Okigami M Ide S Toiyama Y. *In Vivo* Characterization of Neutrophil Extracellular Traps in Various Organs of a Murine Sepsis Model. PloS One (2014) 9(11):e111888. doi: 10.1371/journal.pone.0111888 25372699 PMC4221155

[30] Steppich BA Seitz I Busch G Stein A Ott I. Modulation of Tissue Factor and Tissue Factor Pathway Inhibitor-1 by Neutrophil Proteases. Thromb haemostasis (2008) 100(6):1068–75. doi: 10.1160/TH08-05-0293 19132232

[31] Dwivedi DJ Toltl LJ Swystun LL Pogue J Liaw K-L Weitz JI. Prognostic Utility and Characterization of Cell-Free DNA in Patients With Severe Sepsis. Crit Care (2012) 16(4):R151. doi: 10.1186/cc11466 22889177 PMC3580740

[32] Abrams ST Morton B Alhamdi Y Alsabani M Lane S Welters ID. A Novel Assay for Neutrophil Extracellular Trap Formation Independently Predicts Disseminated Intravascular Coagulation and Mortality in Critically Ill Patients. Am J Respir Crit Care Med (2019) 200(7):869–80. doi: 10.1164/rccm.201811-2111OC PMC6812439 31162936

[33] McDonald B Davis RP Kim SJ Tse M Esmon CT Kolaczkowska E. Platelets and Neutrophil Extracellular Traps Collaborate to Promote Intravascular Coagulation During Sepsis in Mice. Blood (2017) 129(10):1357–67. doi: 10.1182/blood-2016-09-741298 PMC5345735 28073784

[34] Megens RT Vijayan S Lievens D Doring Y van Zandvoort MA Grommes J. Presence of Luminal Neutrophil Extracellular Traps in Atherosclerosis. Thromb haemostasis (2012) 107(3):597–8. doi: 10.1160/th11-09-0650 22318427

[35] Warnatsch A Ioannou M Wang Q Papayannopoulos V. Inflammation. Neutrophil Extracellular Traps License Macrophages for Cytokine Production in Atherosclerosis. Sci (New York NY) (2015) 349(6245):316–20. doi: 10.1126/science.aaa8064 PMC4854322 26185250

[36] Wang F Liu S Pei J Cai L Liu N Liang T. LPA(3)-Mediated Lysophosphatidic Acid Signaling Promotes Postnatal Heart Regeneration in Mice. Theranostics (2020) 10(24):10892–907. doi: 10.7150/thno.47913 PMC7532668 33042260

[37] Birgbauer E Chun J. New Developments in the Biological Functions of Lysophospholipids. Cell Mol Life Sci CMLS (2006) 63(23):2695–701. doi: 10.1007/s00018-006-6155-y PMC11136021 16988788

[38] Anliker B Chun J. Cell Surface Receptors in Lysophospholipid Signaling. Semin Cell Dev Biol (2004) 15(5):457–65. doi: 10.1016/j.semcdb.2004.05.005 15271291

[39] Zhao J He D Su Y Berdyshev E Chun J Natarajan V. Lysophosphatidic Acid Receptor 1 Modulates Lipopolysaccharide-Induced Inflammation in Alveolar Epithelial Cells and Murine Lungs. Am J Physiol Lung Cell Mol Physiol (2011) 301(4):L547–L56. doi: 10.1152/ajplung.00058.2011 PMC3191756 21821728

[40] Li T Peng R Wang F Hua L Liu S Han Z. Lysophosphatidic Acid Promotes Thrombus Stability by Inducing Rapid Formation of Neutrophil Extracellular Traps: A New Mechanism of Thrombosis. J Thromb Haemost (2020) 18(8):1952–64. doi: 10.1111/jth.14839 32291893

[41] Ye X Hama K Contos JJA Anliker B Inoue A Skinner MK. LPA3-Mediated Lysophosphatidic Acid Signalling in Embryo Implantation and Spacing. Nature (2005) 435(7038):104–8. doi: 10.1038/nature03505 PMC1369590 15875025

[42] Meng K Wu B Gao J Cai Y Yao M Wei L. Immunity-Related Protein Expression and Pathological Lung Damage in Mice Poststimulation With Ambient Particulate Matter From Live Bird Markets. Front Immunol (2016) 7:252. doi: 10.3389/fimmu.2016.00252 27446082 PMC4921493

[43] An D Hao F Zhang F Kong W Chun J Xu X. CD14 is a Key Mediator of Both Lysophosphatidic Acid and Lipopolysaccharide Induction of Foam Cell Formation. J Biol Chem (2017) 292(35):14391–400. doi: 10.1074/jbc.M117.781807 PMC5582834 28705936

[44] Yung YC Stoddard NC Chun J. LPA Receptor Signaling: Pharmacology, Physiology, and Pathophysiology. J Lipid Res (2014) 55(7):1192–214. doi: 10.1194/jlr.R046458 PMC4076099 24643338

[45] Inoue M Rashid MH Fujita R Contos JJA Chun J Ueda H. Initiation of Neuropathic Pain Requires Lysophosphatidic Acid Receptor Signaling. Nat Med (2004) 10(7):712–8. doi: 10.1038/nm1060 15195086

[46] Matas-Rico E García-Diaz B Llebrez-Zayas P López-Barroso D Santín L Pedraza C. Deletion of Lysophosphatidic Acid Receptor LPA1 Reduces Neurogenesis in the Mouse Dentate Gyrus. Mol Cell Neurosci (2008) 39(3):342–55. doi: 10.1016/j.mcn.2008.07.014 PMC3667670 18708146

[47] Ong WY Farooqui T Farooqui AA. Involvement of Cytosolic Phospholipase A(2), Calcium Independent Phospholipase A(2) and Plasmalogen Selective Phospholipase A(2) in Neurodegenerative and Neuropsychiatric Conditions. Curr Med Chem (2010) 17(25):2746–63. doi: 10.2174/092986710791859289 20586719

[48] Ishii I Contos JJ Fukushima N Chun J. Functional Comparisons of the Lysophosphatidic Acid Receptors, LP(A1)/VZG-1/EDG-2, LP(A2)/EDG-4, and LP(A3)/EDG-7 in Neuronal Cell Lines Using a Retrovirus Expression System. Mol Pharmacol (2000) 58(5):895–902. doi: 10.1124/mol.58.5.895 11040035

[49] Ishii I Fukushima N Ye X Chun J. Lysophospholipid Receptors: Signaling and Biology. Annu Rev Biochem (2004) 73:321–54. doi: 10.1146/annurev.biochem.73.011303.073731 15189145

[50] Ye X. Lysophospholipid Signaling in the Function and Pathology of the Reproductive System. Hum Reprod Update (2008) 14(5):519–36. doi: 10.1093/humupd/dmn023 18562325

[51] Ahn W-G Jung J-S Song D-K. Lipidomic Analysis of Plasma Lipids Composition Changes in Septic Mice. Korean J Physiol Pharmacol (2018) 22(4):399–408. doi: 10.4196/kjpp.2018.22.4.399 29962854 PMC6019871

[52] Ahn W-G Jung J-S Kwon HY Song D-K. Alteration of Lysophosphatidylcholine-Related Metabolic Parameters in the Plasma of Mice With Experimental Sepsis. Inflammation (2017) 40(2):537–45. doi: 10.1007/s10753-016-0500-6 28028754

[53] Zhao J Wei J Weathington N Jacko AM Huang H Tsung A. Lysophosphatidic Acid Receptor 1 Antagonist Ki16425 Blunts Abdominal and Systemic Inflammation in a Mouse Model of Peritoneal Sepsis. Transl Res (2015) 166(1):80–8. doi: 10.1016/j.trsl.2015.01.008 PMC4458421 25701366

[54] Wright SD Ramos RA Tobias PS Ulevitch RJ Mathison JC. CD14, a Receptor for Complexes of Lipopolysaccharide (LPS) and LPS Binding Protein. Sci (New York NY) (1990) 249(4975):1431–3. doi: 10.1126/science.1698311 1698311

[55] Yang J Nie Y Wang F Hou J Cong X Hu S. Reciprocal Regulation of miR-23a and Lysophosphatidic Acid Receptor Signaling in Cardiomyocyte Hypertrophy. Biochim Biophys Acta (2013) 1831(8):1386–94. doi: 10.1016/j.bbalip.2013.05.005 23711961

[56] Zhang C Li W Lei X Xie Z Qi L Wang H. Targeting Lysophospholipid Acid Receptor 1 and ROCK Kinases Promotes Antiviral Innate Immunity. Sci Adv (2021) 7(38):eabb5933. doi: 10.1126/sciadv.abb5933 34533996 PMC8448453

[57] Cecconi M Evans L Levy M Rhodes A. Sepsis and Septic Shock. Lancet (London England) (2018) 392(10141):75–87. doi: 10.1016/S0140-6736(18)30696-2 29937192

[58] Dejager L Pinheiro I Dejonckheere E Libert C. Cecal Ligation and Puncture: The Gold Standard Model for Polymicrobial Sepsis? Trends Microbiol (2011) 19(4):198–208. doi: 10.1016/j.tim.2011.01.001 21296575

