# Supplementary material for: Lysophosphatidic Acid Receptor 3 Suppress Neutrophil Extracellular Traps Production and Thrombosis During Sepsis
Source: Front Immunol. 2022 Apr 7;13:844781. doi: 10.3389/fimmu.2022.844781 (PMC9021375; doi:10.3389/fimmu.2022.844781)
Supplement: Supplementary file 1 [file DataSheet_1.pdf]

Supplementary Material

1 Supplementary Figures and Tables

1.1 Supplementary Tables

Table 1: List of primer sequences used for mice genotype identification and qRT-PCR

Primer name	Sequence information (5' to 3')	Use
LPA_3 genotypes- e1b	TGACAAGCGCATGGACTTTTTC	genotype identification
LPA_3 genotypes-e1c	GAAGAAATCCGCAGCAGCTAA	genotype identification
LPA_3 genotypes-New F	GCACGAGACTAGTGAGACGTGCTAC	genotype identification
CD14-Forward	CTCTGTCCTTAAAGCGGCTTAC	qRT-PCR
CD14-Reverse	GTTGCGGAGGTTCAAGATGTT	qRT-PCR
IL-6-Forward	CCAAGAGGTGAGTGCTTCCC	qRT-PCR
IL-6-Reverse	CTGTTGTTCAGACTCTCTCCCT	qRT-PCR
IL-8-Forward	CAAGGCTGGTCCATGCTCC	qRT-PCR
IL-8-Reverse	TGCTATCACTTCCTTTCTGTTGC	qRT-PCR
GAPDH-Forward	TTGCACGTTACACTGCTTGC	qRT-PCR
GAPDH-Reverse	GTGGTCATGAGCCCTTCCA	qRT-PCR

1.2 Supplementary Figures

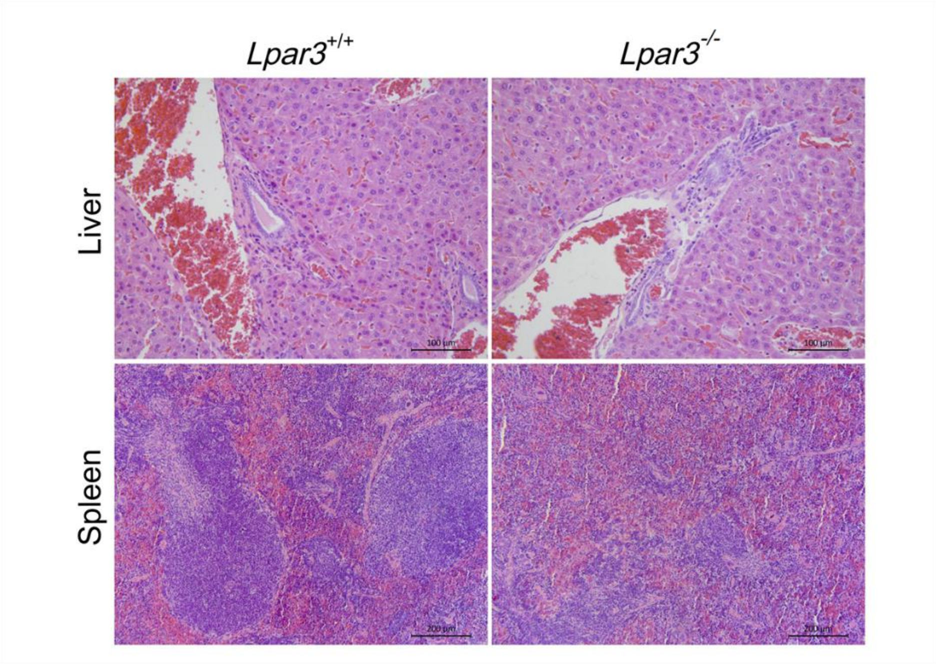

Supplementary Figure 1. Hematoxylin and eosin staining of the liver and spleen of sepsis mice. *Lpar3*$^{-/-}$ sepsis mice showed significant hyperplasia of the splenic red marrow. Bar = 200μm for liver Bar = 200 μm for spleen.

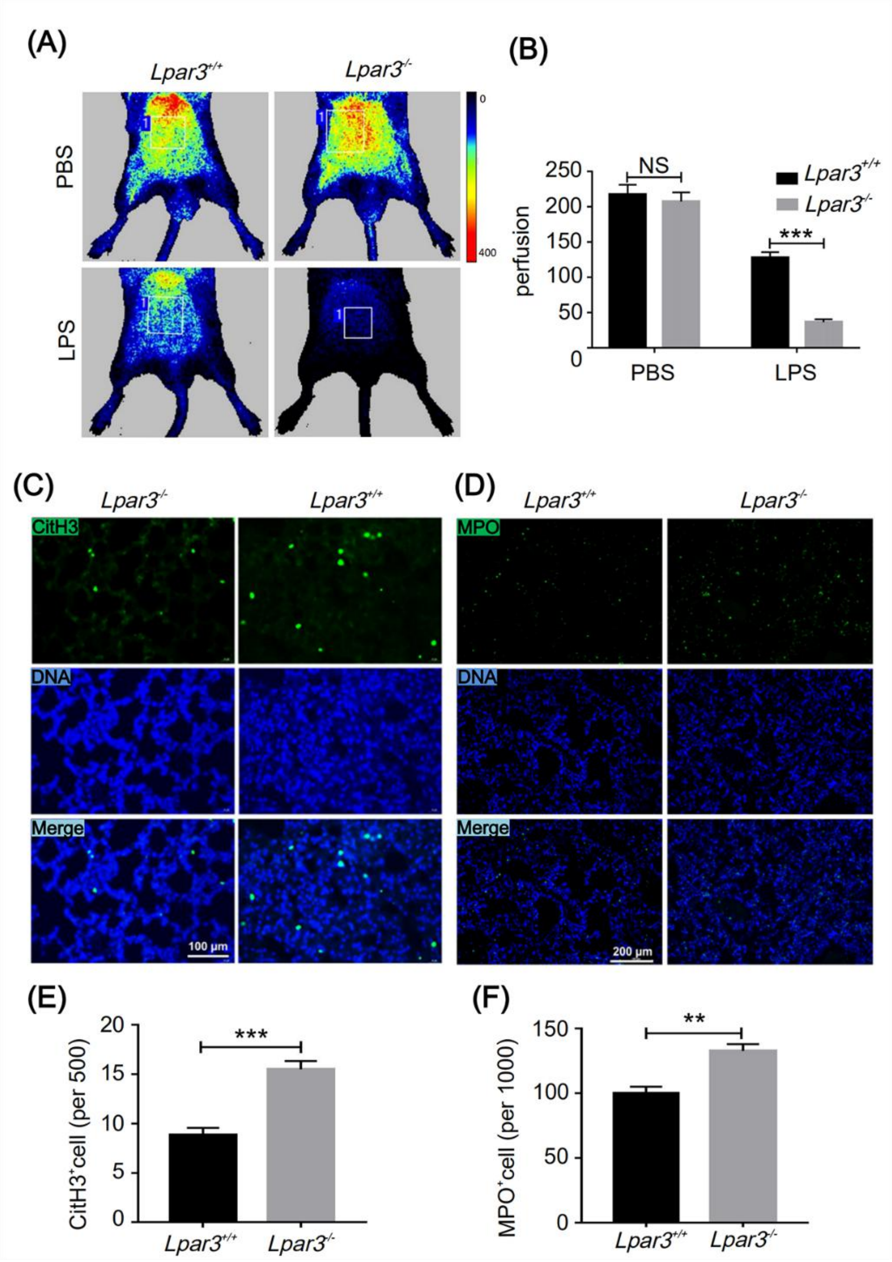

Supplementary Figure 2. Blood flow tests of sepsis mice and immunofluorescence staining of NETs in the lungs of sepsis mice. (A, B): Blood flow tests of sepsis mice showed that *Lpar3*$^{-/-}$ sepsis mice had almost no blood flow in the body skin, and microcirculation was seriously blocked. n = 5 for *Lpar3*$^{+/+}$ or *Lpar3*$^{-/-}$ controls, n = 8 for *Lpar3*$^{+/+}$ LPS, n = 7 for *Lpar3*$^{-/-}$ LPS; **(C-F):** Immunofluorescence assays for NETs markers in lung tissue confirmed that the expression of $CitH_3$ and MPO were much higher in *Lpar3*$^{-/-}$ mice than in WT controls after LPS injection (n = 4 for each group). ** $P < 0.01$, *** $P < 0.0001$, NS, not significance. Abbreviations: LPS, lipopolysaccharide; LPA, lysophosphatidic acid; LPA_3, lysophosphatidic acid receptor 3

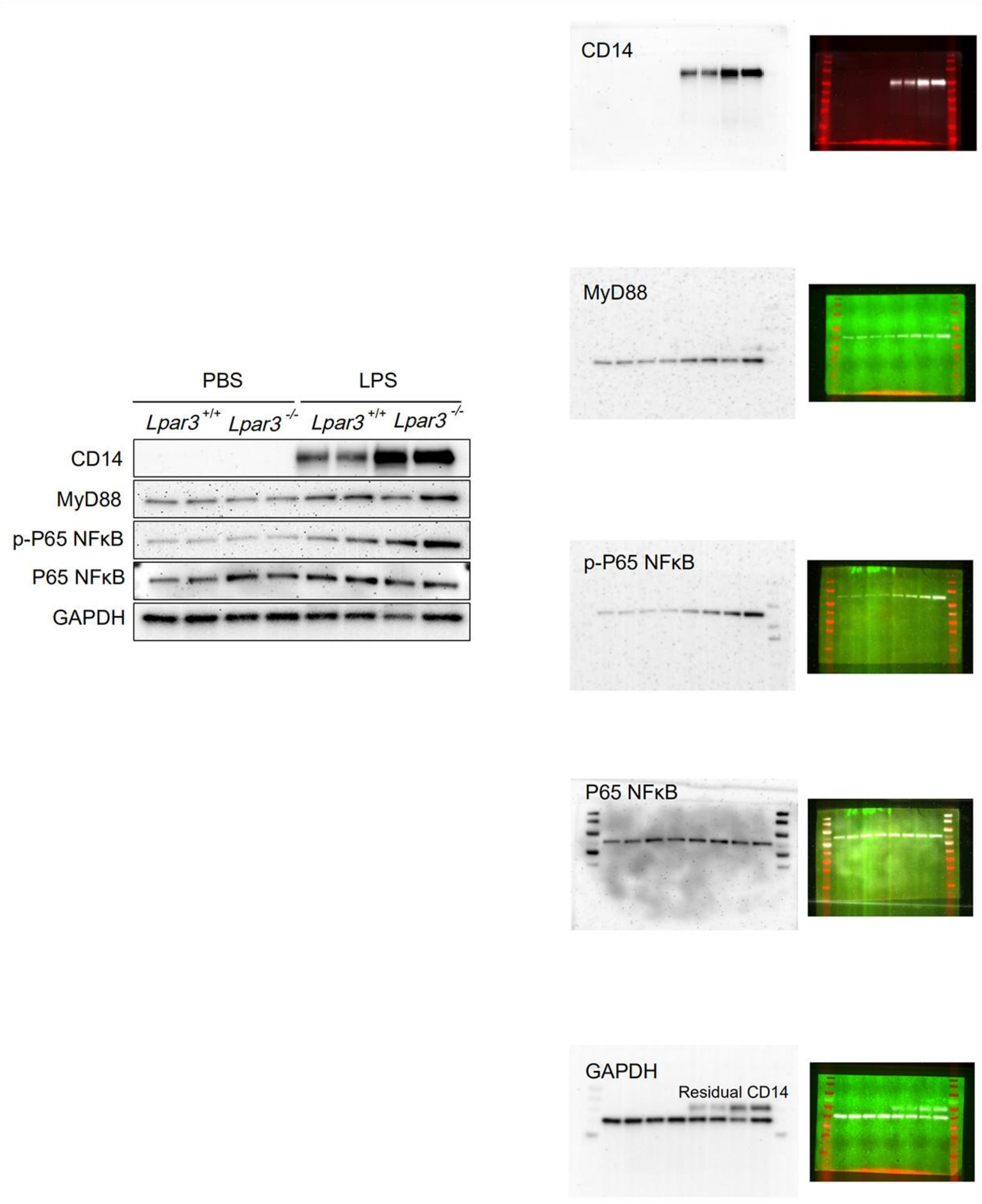

Supplementary Figure 3. Merge image and images of each protein hybridization membrane.

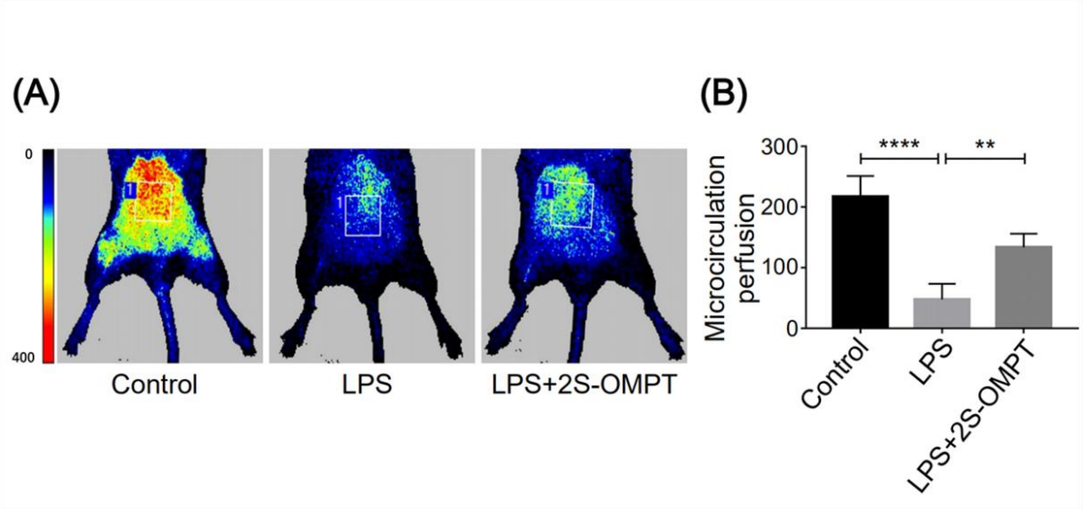

Supplementary Figure 4. Blood flow tests of sepsis mice with (2S)-OMPT. After (2S)-OMPT injection, epidermal blood flow was restored, and the degree of microcirculatory embolism was significantly reduced in sepsis mice. n = 6 for control, n = 7 for LPS + BSA, n = 6 for LPS+(2S)-OMPT); **P < 0.01; ****P < 0.00001. Abbreviations: LPS, lipopolysaccharide; (2S)-OMPT, (2S)-1-oleoyl-2-methylglycero-3-phosphothionate.
